# Whole-body galactose oxidation as a robust functional assay to assess the efficacy of gene-based therapies in a mouse model of Galactosemia

**DOI:** 10.1016/j.omtm.2024.101191

**Published:** 2024-01-17

**Authors:** Bijina Balakrishnan, Xinhua Yan, Marshall D. McCue, Olivia Bellagamba, Aaron Guo, Felicity Winkler, Jason Thall, Lisa Crawford, Rain Dimen, Sara Chen, Sean McEnaney, Yiman Wu, Mike Zimmer, Joe Sarkis, Paolo G.V. Martini, Patrick F. Finn, Kent Lai

**Affiliations:** 1 Division of Medical Genetics, Department of Pediatrics, University of Utah School of Medicine, Salt Lake City, UT 84108, USA; 2 Moderna, Cambridge, MA 02139, USA; 3 Sable Systems International, North Las Vegas, NV 89032, USA

**Keywords:** classic galactosemia, gene therapy, mRNA-based therapy, breath test, galactose oxidation, adeno-associated viral vectors, lipid nanoparticles

## Abstract

Despite the implementation of lifesaving newborn screening programs and a galactose-restricted diet, many patients with classic galactosemia develop long-term debilitating neurological deficits and primary ovarian insufficiency. Previously, we showed that the administration of human *GALT* mRNA predominantly expressed in the *GalT* gene-trapped mouse liver augmented the expression of hepatic GALT activity, which decreased not only galactose-1 phosphate (gal-1P) in the liver but also peripheral tissues. Since each peripheral tissue requires distinct methods to examine the biomarker and/or GALT effect, this highlights the necessity for alternative strategies to evaluate the overall impact of therapies. In this study, we established that whole-body galactose oxidation (WBGO) as a robust, noninvasive, and specific method to assess the *in vivo* pharmacokinetic and pharmacodynamic parameters of two experimental gene-based therapies that aimed to restore GALT activity in a mouse model of galactosemia. Although our results illustrated the long-lasting efficacy of AAVrh10-mediated *GALT* gene transfer, we found that *GALT* mRNA therapy that targets the liver predominantly is sufficient to sustain WBGO. The latter could have important implications in the design of novel targeted therapy to ensure optimal efficacy and safety.

## Graphical abstract

**Figure:**
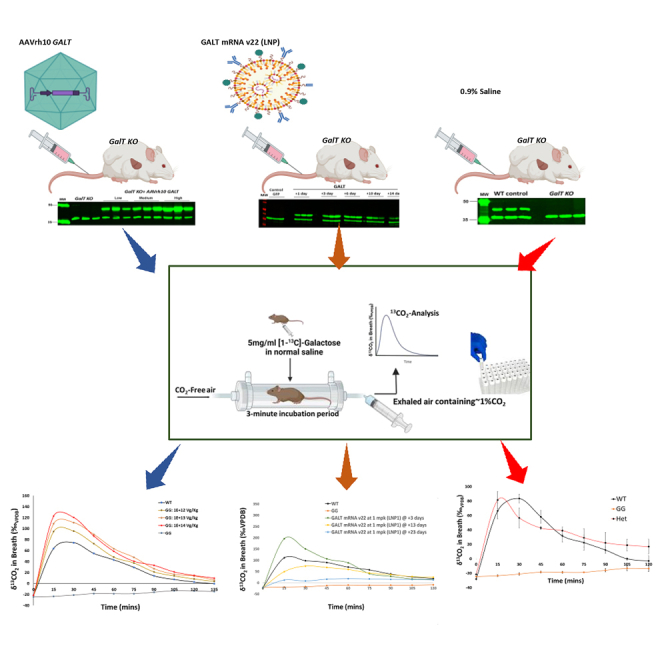

## Teaser

Lai and colleagues established whole-body galactose oxidation (WBGO) as a robust, noninvasive, and specific method to assess the *in vivo* pharmacokinetic and pharmacodynamic parameters of two experimental gene-based therapies (GALT mRNA and AAVrh10-*GALT* gene replacement) that aimed to restore GALT activity in a mouse model of Galactosemia.

## Introduction

Classic galactosemia (CG) (OMIM: 230400) is an autosomal recessive disorder caused by the deficiency of galactose-1-phosphate uridylyltransferase (GALT, EC 2.7.7.12) activity.^1^^,^^2^^,^^3^^,^^4^^,^^5^ GALT is the second enzyme in the evolutionarily conserved galactose metabolic pathway, and it facilitates the simultaneous conversion of uridine diphosphoglucose (UDP-glucose) and galactose-1 phosphate (gal-1P) to uridine diphosphogalactose (UDP-galactose) and glucose-1 phosphate.^6^ Consequently, GALT deficiency leads to the accumulation of gal-1P and deficiency of UDP-galactose in patient cells.^7^^,^^8^ If untreated, CG can be lethal for affected newborns.^1^^,^^5^ Since the inclusion of this disease in the newborn screening panel in the United States, neonatal mortality has decreased.^9^ The mainstay of treatment is the withdrawal of galactose from the diet.^5^ However, despite early and adequate dietary management, endogenous production of galactose/gal-1P persists,^10^^,^^11^ and many patients suffer long-term complications such as intellectual deficits in ≥6-year-olds (45% of patients), speech delay in ≥3-year-olds (56%), motor function deficits (tremors and cerebellar ataxia) in ≥5-year-olds (18%), and primary ovarian insufficiency (POI) (91%).^12^^,^^13^ Decreased bone mineralization is increasingly recognized in prepubertal patients of either sex.^14^^,^^15^^,^^16^ Except for POI, which is nearly universal among affected females, there is great variability in manifestation among other long-term complications. Some attribute the variability to epigenetic factors, but none have been convincingly identified. Although aberrant galactosylation of glycoproteins/lipids and inositol phospholipid signaling caused by chronic accumulation of toxic intermediates of the blocked galactose metabolic pathway have been proposed,^17^^,^^18^^,^^19^^,^^20^^,^^21^^,^^22^ the environmental and molecular mechanisms for these long-term complications remain enigmatic. There are currently no satisfactory treatments available to prevent/alleviate any of these complications. Regardless of the molecular pathophysiological mechanisms, the root cause of the disease is the deficiency of GALT enzyme activity in patient cells. Consequently, therapeutic strategies that aim to restore the GALT enzyme activity in patients are being explored, and among those, experimental mRNA therapy and adeno-associated virus (AAV)–based gene replacement therapies have been previously reported.^23^^,^^24^ Although these experimental therapies demonstrated exceptional efficacy in normalizing the disease-relevant biomarkers in the target organs such as liver in animal models, the degree of normalization has been modest in red blood cells (RBCs), likely due to limited protein synthesis in the non-nucleated RBC and/or inefficient delivery of the *GALT* mRNA/cDNA. Therefore, if the remedial alterations in organs such as the liver or brain are not reflected in the corresponding analyses in the RBCs, there will be a need for an alternative method to assess treatment efficacy. In this study, we established the use of whole-body galactose oxidation as such an alternative method to evaluate the *in vivo* pharmacokinetic and dose-dependent pharmacodynamic parameters of two experimental gene-based therapies that aimed to restore GALT activity in a mouse model of CG.

## Results

### GalT-deficient mice cannot metabolize injected ^13^C-galactose to ^13^CO_2_

**Figure 1:**
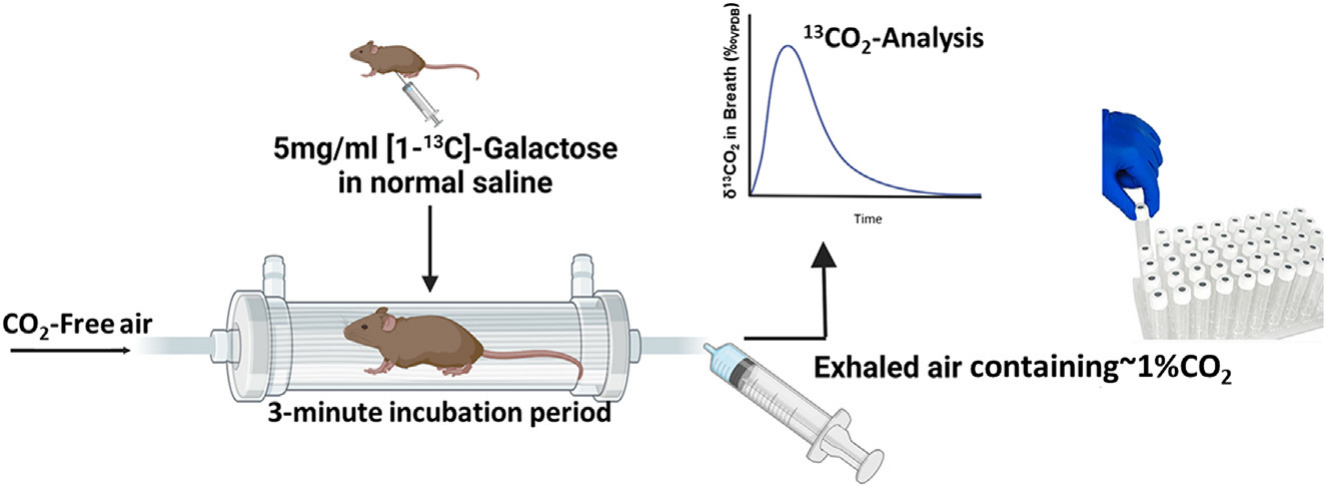
Schematic representation of breath test analysis After injection with ^13^C-galactose, the animal is quickly relocated to the air-sealed chamber. Expired air was collected using a 20-mL glass syringe every 15 min over 2 h. A tank of CO_2_-free air was used to flush out the chamber for 10 s before subsequent samples. The collected air samples were stored in a Exetainer vial and the analyses of ^13^CO_2_ enrichment used a published protocol.

Patients with CG (GALT deficiency) have repeatedly been shown to be incapable of oxidizing ^13^C-galactose *in vivo* to ^13^CO_2_ in the breath.^25^^,^^26^^,^^27^^,^^28^^,^^29^^,^^30^ Therefore, if we want to test the efficacy of the gene-based therapy in our GalT-deficient mice using the same whole-body galactose oxidation test, we will have to show that the mutant mice perform similarly to human patients. As described in materials and methods, we challenged wild-type (WT) and the GalT-deficient (GG) mice with an intraperitoneal (i.p.) dose of ^13^C-galactose and monitored the enrichment of ^13^CO_2_ in the breath for 120 min using the apparatus setup depicted in Figure 1.

**Figure 2:**
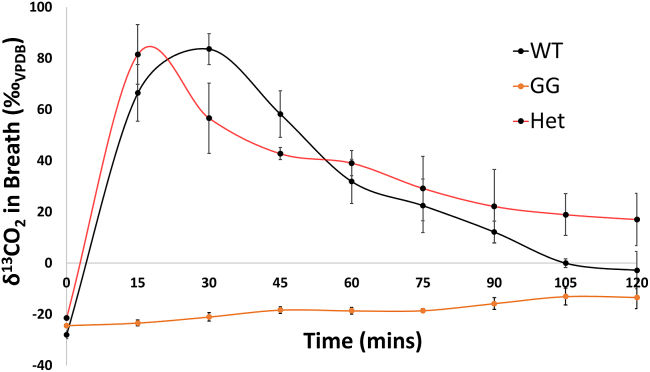
Evaluation of whole-body ^13^C-galactose oxidation in WT, homozygous *GalT-*KO (GG), and heterozygous *GalT*-KO (Het) mice After i.p. administration of 5 mg [1-^13^C] galactose, the amount of ^13^CO_2_ in the expelled air over time was measured and calculated as described in materials and methods.

As shown in Figure 2, both WT and heterozygous mice were able to oxidize the labeled galactose to ^13^CO_2_ almost instantaneously, with the maximum enrichment level reached between 20 and 30 min postinjection. Thereafter, ^13^C enrichment gradually declined over the subsequent 100 min. The cumulative galactose oxidation (as measured by the area under the curve) was statistically higher in the WT (Tukey honestly significant difference [HSD]; p = 0.012) and heterozygous mice (p = 0.018) when compared to the GalT-deficient (GG) mutants, In fact, no galactose oxidation was detected in the GalT-deficient (GG) mutants throughout the entire test duration.

### Reexpression of GALT activity by experimental AAVrh10-*GALT* gene replacement in GalT-deficient mice restores whole-body ^13^C-galactose oxidation

**Figure 3:**
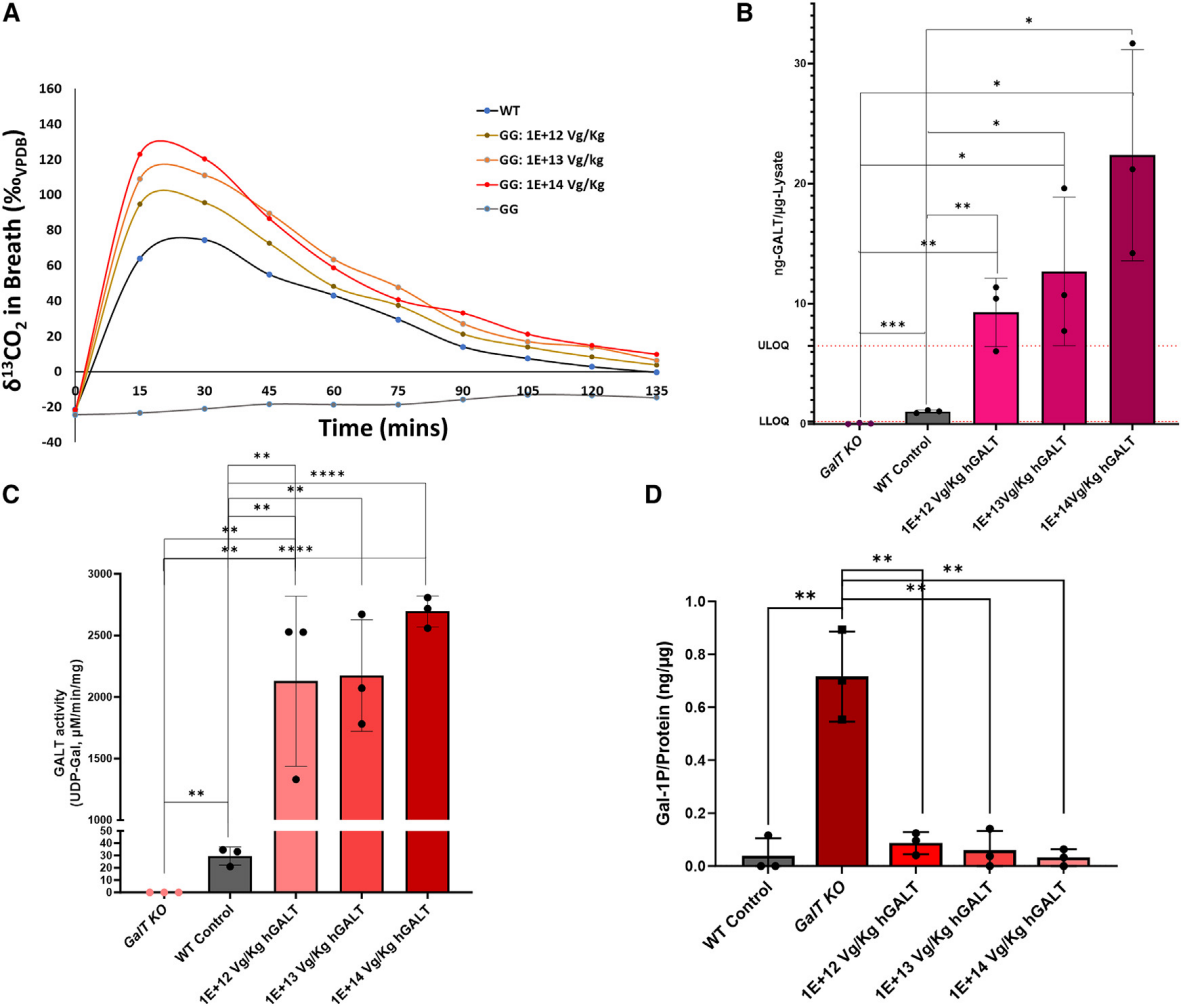
Dose dependency of whole-body ^13^C-galactose oxidation in GalT-deficient mice after experimental AAVrh10 *GALT* gene replacement therapy (A) Four-week-old *GalT* KO male mice were injected with a single i.p. dose of AAVrh10 *GALT* at a dose of 1 × 10^12^, 1 × 10^13^, and 1 × 10^14^ vg/kg body weight, respectively (n = 3 per group). Seven days after the injection, the amount of ^13^CO_2_ in the expelled air over time was measured and calculated as described in materials and methods. Curves are interpolated using a “cubic spline.” (B and C) Specific GALT protein quantification (B) and (C) Specific GALT enzyme activity analysis in *GalT*-KO and AAVrh10-*GALT*-treated mice. (D) The comparison of the disease-relevant biomarker gal-1P in liver samples of *GalT*-KO and AAVrh10-*GALT*-treated mice. Values presented as mean ± SD. (∗p < 0.05; ∗∗p < 0.005; ∗∗∗p < 0.0005; ∗∗∗∗p < 0.0001).

To test the hypothesis that whole-body galactose oxidation can be used as a functional assay for the effectiveness of AAV-based *GALT* gene replacement therapy, cohorts (n = 3 or 4) of GG mice were injected with three different dosages of the AAVrh10-*GALT* vector, which allow high *GALT* gene expression under the CAG promoter in the infected cells. Figure 3A shows that the mutant mice regained their whole-body galactose oxidation capacity in a dose-dependent manner 7 days after AAVrh10-*GALT* vector injection (Pearson correlation; p = 0.002). Due to the strong CAG promoter, it is not surprising that even at the lowest dosage, the maximum level of galactose oxidation was 28.5% higher than that of WT. The maximum level of galactose oxidation was 65.2% higher of that of WT in the highest dosage.

To further illustrate the relevance of whole-body galactose oxidation in assessing the efficacy of gene-based therapies in replenishing GALT activity and normalization of biomarkers in disease-relevant organs that are not easily accessible in human patients, we chose to correlate the whole-body galactose oxidation results (Figure 3A) with GALT expression in the livers of the knockout (KO) mice 9 weeks after injection with the various dosages. When we examined the levels of GALT protein expressed in the livers of different groups of animals, we showed that the level of expressed GALT protein in the treated KO mice liver was ∼10-fold that of WT at the lowest dosage, and ∼25-fold of that of WT at the highest dosage, as determined by specific protein quantification (Figure 3B) and GALT activity determination (Figure 3C). The high level of expression of GALT activity was accompanied by the normalization of the disease-relevant biomarker gal-1P in the liver (Figure 3D). Statistical analyses showed that all three dosages result in significant increases in GALT protein and GALT activity compared to untreated GG and WT mice (Figures 3B–3D). Overall, the results largely mirror the whole-body galactose oxidation data shown in Figure 3A and corroborate the dose-dependent responses described below.

### Replenishment of hepatic GALT activity by experimental *GALT* mRNA therapy in GalT-deficient mice restores whole-body ^13^C-galactose oxidation

**Figure 4:**
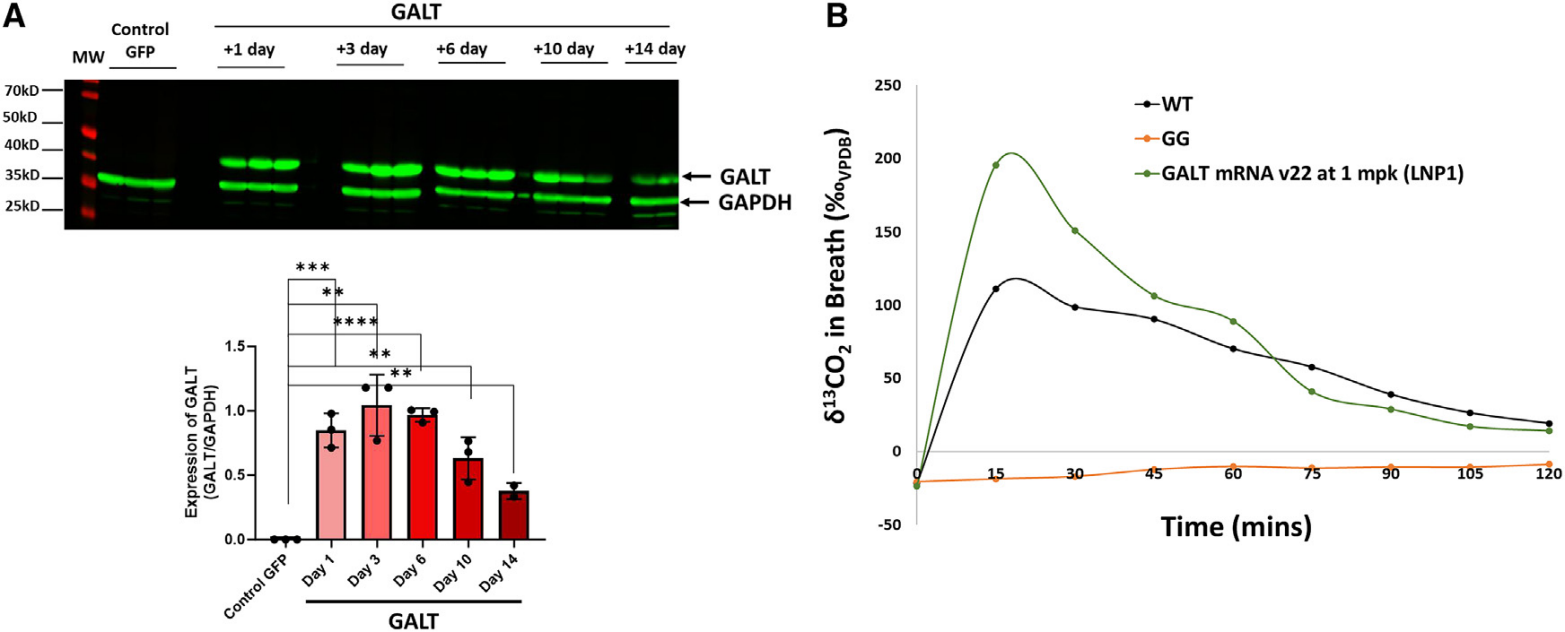
Single iv dose of LNP1-encapsulated *GALT* mRNA version 22 resulted in long half-lived GALT protein and normalized whole-body galactose oxidation (A) Kinetic analysis of liver GALT protein using different cohorts of *GalT*-KO mice after a single dose of 1 mpk *GALT* mRNA version 22. (B) Whole-body galactose oxidation was measured in the treated animals 3 days after treatment. (∗∗p < 0.005; ∗∗∗p < 0.0005; ∗∗∗∗p < 0.0001).

With the above promising results, we expanded our investigation to another gene-based approach that holds significant promise as a form of novel therapy—experimental *GALT* mRNA therapy.^23^ Previously, we showed that intravenous (i.v.) injection of lipid nanoparticle 1 (LNP1)–encapsulated *GALT* mRNA restores normal *GALT* expression and galactose metabolism in the liver of our *GalT* gene-trapped mouse model.^23^ In this study, we wanted to extend our previous findings to see whether *GALT* mRNA, which was predominantly expressed in the liver, restores whole-body galactose oxidation in the animals. However, instead of using the old version of *GALT* mRNA, which has a half-life of ∼3 days,^23^ we used a new optimized version of *GALT* mRNA (*GALT* mRNA version 22) in this study. As shown in Figure 4A, *GALT* mRNA version 22 has an estimated half-life of 9–10 days in the liver. Moreover, a single i.v. dose of *GALT* mRNA version 22/LNP1 at 1 mg/kg resulted in a 24% increase in whole-body galactose oxidation at 3 days post-mRNA therapy (Figure 4B).

### Whole-body ^13^C-galactose oxidation can be used to monitor the sustainability of experimental *GALT* gene-based therapies over time

**Figure 5:**
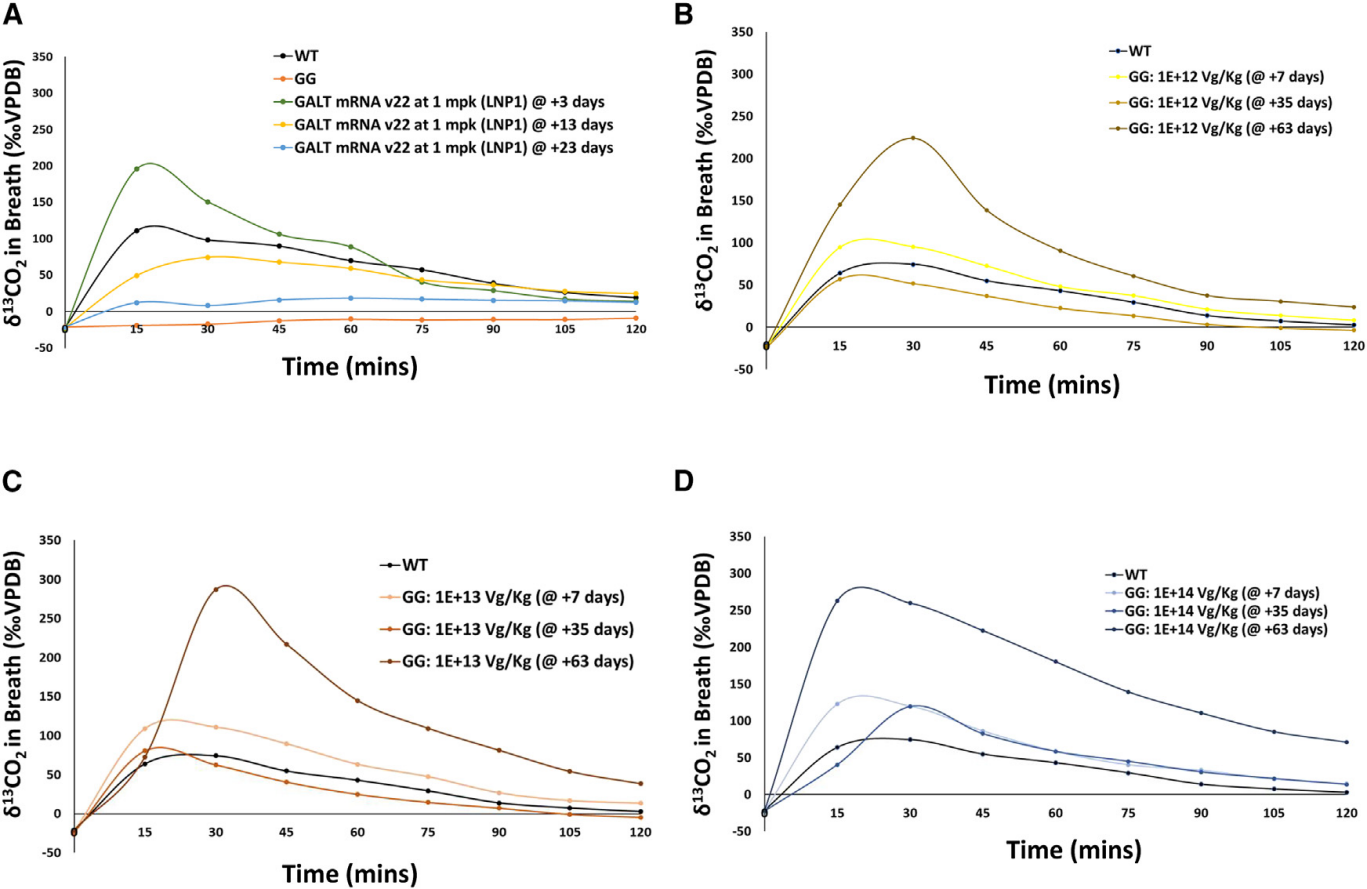
Time course analyses of whole-body galactose oxidation in *GalT* KO mice treated with LNP1-encapsulated *GALT* mRNA version 22 therapy or AAVrh10-*GALT* gene therapy (A) Time course analyses of whole-body galactose oxidation with a single dose (1 mpk) of *GALT* mRNA version 22 in the same cohort of *GalT-*KO mice. (B–D) Time course analyses of whole-body galactose oxidate in the same cohorts of *GalT*-KO mice treated with 3 different doses (1 × 10^12^, 1 × 10^13^, and 1 × 10^14^ vg/kg body weight) of the experimental AAVrh10 *GALT* gene therapy.

The duration of transgene expression in disease-relevant organs for some gene-based therapies such as mRNA therapy and AAV-based gene replacement therapy has significant implications for their long-term therapeutic effectiveness. To see whether whole-body galactose oxidation is a reliable functional biomarker to monitor the expression of the *GALT* gene delivered by either the AAVrh10 *GALT* vector or *GALT* mRNA version 22, we have followed cohorts of treated animals for an extended period after treatments. In Figure 5A, a single administration of GALT mRNA version 22/LNP1 resulted in a whole-body galactose oxidation response that was an average of 118% of the WT level on day 3. Thereafter, we observed a time-dependent decrease over the subsequent 20 days (regression ANOVA, p = 0.015). This response was sustained at 35% of the WT level up until day 23.

AAVrh10 *GALT*-treated KO animals maintained substantial galactose oxidation up to 5 weeks (+35 days) after treatment across doses that ranged three magnitudes of order (Figures 5B–5D). Surprisingly, by day +63, galactose oxidation capacity increased to 216% of that seen in WT mice across all three doses examined. (Figures 5B–5D).

### Enhancing whole-body galactose oxidation: Exploring the use of a different LNP class with *GALT* mRNA version 22

**Figure 6:**
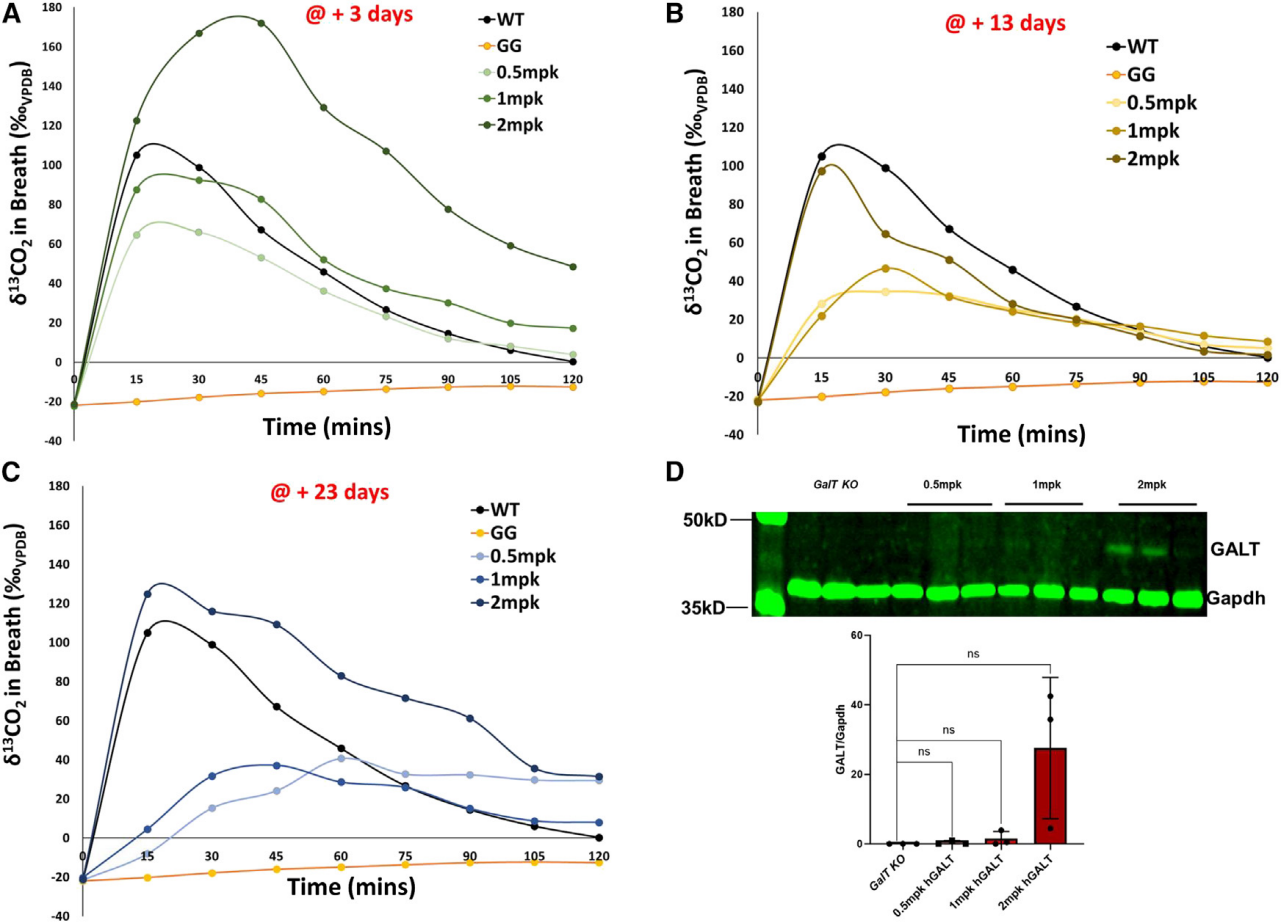
Time course analyses of whole-body galactose oxidation in *GalT* KO mice treated with LNP4-encapsulated *GALT* mRNA version 22 therapy (A–C) Time course analyses of whole-body galactose oxidation with a single, but 3 different dosages (0.5, 1, and 2 mpk) of *GALT* mRNA version 22 in the same cohorts of *GalT-*KO mice. (D) Western blot analysis of GALT expression in the animals 23 days after treatment. Values presented as mean ± SD.

The data shown in Figures 4 and 5A were obtained using LNP1, which was discovered in 2018.^31^ We intended to investigate *GALT* mRNA version 22 in a novel designed LNP (LNP4) to evaluate its effect on the expression of *GALT* mRNA version 22 as measured by whole-body galactose oxidation. As shown in Figure 6A, we found that LNP4-encapsulated *GALT* mRNA version 22 showed a dose-dependent response (Pearson correlation, p = 0.011). At +3 days, 1 mpk was sufficient to induce whole-body galactose oxidation similar to that seen in WT mice. Increasing the dosage to 2 mpk increased significantly the level of whole-body galactose oxidation at day +3 (∼2-fold WT level). Significant dose dependence of whole-body galactose oxidation was not detected at day +13 of mRNA administration (Figure 6B; Pearson correlation; p = 0.119), but it was reestablished by day +23 (Figure 6C; p = 0.029). In addition, 23 days after mRNA administration, we assessed the GALT expression in the livers of the KO mice. As seen in Figure 6D, 2 mpk *GALT* mRNA results in a higher amount of GALT expression.

## Discussion

Recent advances in molecular therapeutics and gene-based vaccines have paved the way for new treatment modalities for monogenic diseases such as phenylketonuria and CG. Among these modalities, gene replacement therapies and mRNA-based therapies are gaining popularity because, if successful, these modalities will address the root cause of the diseases—the absence of functional gene products—thus making them attractive and rational choices. Although efficient vectors and nanoparticles have been developed to deliver the cDNA and mRNA, respectively, to the disease-relevant organs, the *in situ* pharmacokinetics (PK) and pharmacodynamics (PD) assessments for the specific modality could be challenging clinically if the organs of interest are inaccessible for repeated sampling. Consequently, surrogate disease-relevant biomarkers could prove useful for part of the portfolio for the evaluation of effectiveness of the treatments under these circumstances. Ideally, the desired biomarkers are those directly implicated in the pathogenic mechanisms of the diseases so that their changes can truly reflect the change in disease states and phenotypes. However, the underlying pathophysiological mechanisms of many diseases are often not fully elucidated, which further complicates the identification of disease-relevant biomarkers for the evaluation of therapeutic efficacy.

In this study, we tested the hypothesis that whole-body galactose oxidation can be used as a noninvasive, robust, accurate, and functional biomarker for testing the effectiveness of gene-based therapies for CG. In CG, patients suffer a block in the galactose metabolic pathway because of the inherited deficiency of the enzyme GALT.^1^^,^^2^^,^^3^^,^^4^^,^^5^ Galactosemia patients suffer from a host of neurological complications such as ataxia, and in females, POI.^12^^,^^32^^,^^33^ For decades, diagnosis of the disease relied on the detection of the abnormal accumulation of galactose metabolites such as RBC gal-1P, as well as the absence of RBC GALT activity.^34^ This protocol works well for diagnosis because normal RBCs have detectable GALT expression.^35^^,^^36^ However, when it comes to gene-based therapies that are aimed to restore normal GALT activity in disease-relevant tissues, the *in situ* PK/PD assessments become challenging because the affected organs in galactosemia (i.e., liver, brain, and ovary) are not amendable to repeated sample collections. In addition, the vectors/nanoparticles used to deliver the cDNA/mRNA may not efficiently target RBCs, the cell type that is typically used for PK/PD evaluation. Last but not least, the reduction/reversal of the accumulation of galactose metabolites in the blocked galactose metabolic pathway is an indirect and rather nonspecific measure of genetic restoration of GALT activity. For instance, it has been shown that the administration of aldose reductase can also reduce accumulation of plasma galactitol in patients and experimental animals,^37^^,^^38^ even when normal galactose metabolism is not restored in the disease-relevant organs, the common goal for *GALT* gene-based therapies. Consequently, we hypothesize that whole-body galactose oxidation is a promising alternative because it is noninvasive, and it measures the restoration of galactose metabolism by examining the direct function of the reconstituted *GALT* expression. More important, whole-body galactose oxidation has been performed in galactosemic patients, and their *in vivo* capacity to oxidize galactose correlates with their long-term outcome.^30^^,^^39^ Furthermore, whole-body substrate oxidation assay has been advocated as a reliable method to measure the efficacy of gene-based therapy in other genetic diseases such as methylmalonic acidemia.^40^^,^^41^ Before we can use this test to assess *GALT* gene-based therapies in human patients, we will have to validate this method in animal models.

To begin, we established the experimental conditions at which we could distinguish normal and absent galactose oxidation capacity in WT and *GalT*-KO animals, respectively (Figure 1). The results were similar to what has been done in human patients.^27^ Notably, we saw normal galactose oxidation capacity in heterozygous animals (Figure 2), which is not surprising because heterozygous patients are phenotypically normal in this autosomal recessive disorder.^1^ Another important feature for the test is that the interindividual variability among different animals was relatively small, further indicating that it is a robust and reliable assay.

After setting up the experimental conditions for the oxidation test, we measured the effectiveness of the AAVrh10-mediated *GALT* gene replacement therapy. Experimental AAV vector-based *GALT* gene replacement therapies in animal models have been reported,^24^^,^^42^ but none have used AAVrh10. In addition, the PK/PD assessments of the experimental therapy in the disease-relevant organs in those studies were performed using organs harvested posteuthanasia, underscoring the need for a practical, noninvasive means for clinical evaluation. Our results revealed a dose-dependent response in whole-body galactose oxidation in *GalT*-KO mice injected with increasing quantities of AAVrh10-*GALT* vector (Figure 3A) shortly (7 days) after injection. As shown in Figure 5, we extended the oxidation test to monitor the animals at later time points. These results are significant because they exemplified the use of whole-body galactose in following any change in effectiveness of a therapy over time in the same animal/subject. In fact, if we had not done this, we would not have realized that the whole-body galactose oxidation capacity of the treated animals increased further at +63 days for all doses (Figures 5B–5D). At the moment, we do not know the precise mechanisms for the increase in *GALT* expression at a later time, but we do not think it is an artifact because this was detected with the 3 independent and different doses (i.e., low, medium, and high).

Similar to AAV vector-mediated *GALT* gene replacement, we showed in Figure 4B that we could use whole-body galactose oxidation test to evaluate the expression of *GALT* mRNA version 22 delivered using LNP1. In addition, we showed that *GALT* expression in the liver rendered by the specified dosing regimen is sufficient to yield higher galactose oxidation capacity than normal mice. This is not too surprising because liver is a major organ for galactose metabolism. These findings could have a profound impact on future therapeutic approaches for the disease. As expected with mRNA therapy, we observed a decline in whole-body galactose oxidation capacities in mice at later times, in contrast to those treated with AAVrh10-*GALT* gene replacement (Figure 5A). These outcomes are particularly noteworthy because they confirm the utility of the galactose oxidation test in tracking both the increase and decrease in *GALT* gene expression over time.

Lastly, we used the whole-body galactose oxidation test to compare the efficacies of equal dosage of *GALT* mRNA version 22 encapsulated in two different lipid nanoparticles (LNP1 versus LNP4). Although LNP1 showed higher oxidation capacity in the near term, LNP4 encapsulated mRNA demonstrated longer sustainability (Figures 6A–6D). The performance of two different LNP formulations can vary significantly, often due to their distinct distribution profiles postdelivery. For instance, one LNP may demonstrate superior efficacy in delivering its mRNA payload to liver cells, resulting in a more pronounced therapeutic effect and whole-body galactose oxidation. This could be attributed to its specific lipid composition, which allows for efficient uptake by hepatocytes. However, another LNP may exhibit a broader distribution profile, reaching a variety of tissues. This could lead to a more systemic effect, with more sustained galactose oxidation. These differences underscore the importance of carefully considering the distribution profile when designing and selecting LNP formulations for specific therapeutic applications.

In conclusion, we have demonstrated the vast utility potentials of the whole-body galactose oxidation test in the evaluation of experimental therapies that aim to restore galactose metabolism. Although we are encouraged by the data, we are aware of the lack of linear response between the level of RBC *GALT* expression and whole-body galactose oxidation (see normal oxidation in heterozygous mice in Figure 2). However, we do not see it as a limitation. Instead, we think this further demonstrates that this functional test is a good representation of the disease phenotype because patients who have GALT gene variants that give as low as 10% GALT activity are phenotypically normal under a galactose-restricted diet.^29^

## Materials and methods

### mRNA and LNP synthesis and formulation

mRNA was synthesized and formulated in LNPs as described previously.^23^ The same biodegradable, ionizable LNP described in our previous studies was used in the current set of studies. Briefly, mRNA was synthesized *in vitro* by T7 RNA polymerase-mediated transcription with 5-methoxy uridine-5′-triphosphate(5-methoxyUTP) in place of UTP. The linearized DNA template contains the 5′ and 3′ UTRs, open reading frame, and the poly(A) tail. The mRNA was produced with cap1 to improve translation efficiency. After purification, the mRNA was diluted in 50 mM sodium acetate (pH 5) and mixed with lipids dissolved in ethanol (50:10:38.5:1.5; ionizable: helper:structural:polyethyleneglycol) at a ratio of 3:1 (aqueous:ethanol). The final product was filtered through a 0.22-μm filter and stored in presterilized vials that were frozen until use. The mRNAs were tested for purity and capping efficacy and were found to be >70% and >90%, respectively. All of the formulations were tested for particle size, RNA encapsulation, and endotoxin and were found to be <100 nm in size, with >80% encapsulation, and <10 endotoxin units/mL endotoxin.

### AAV vector

The recombinant vector was designed by the Lai lab and subsequently synthesized at VectorBuilder (Chicago, IL) on a fee-for-service basis. The codon-optimized full-length human *GALT* cDNA was inserted in a multiple cloning site following a cytomegalovirus enhancer and a chicken β-actin promoter, which was used to drive the GALT cDNA.

### Animal model

All of the animal protocols and procedures were approved and conducted in full compliance with the guidelines outlined in the Guide for the Care and Use of Laboratory Animals and were approved by the University of Utah Institutional Animal Care and Use Committee. Galt-deficient mice used in this study were constructed as previously described and were always fed with normal chow after weaning. Mice were housed in standard laboratory cages within temperature (22°C–23°C)- and humidity (30%–70%)-controlled rooms under a 12:12 light:dark cycle. All of the mice were confirmed by genotyping (molecular and biochemical) using a previously published protocol.^43^ Both males and females were used for the present study and were uniformly distributed among each experiment group.

### *In vivo GALT* mRNA administration

Four-week-old GalT-deficient mice were injected i.v. with 100 μL of sterile LNPs provided by Moderna (Cambridge, MA). Three mice per group received multiples dosages at (0.5, 1, and 2 mg/kg) and were used to measure the labeled galactose oxidation at 3, 13, and 23 days after mRNA administration. LNPs were diluted in sterile PBS at specified concentrations. Liver samples were collected and snap-frozen at each time point for GALT protein expression and enzymatic activity.

### *In vivo* AAVrh-10 *GALT* gene administration

Four-week- old homozygous Galt-deficient mice were treated via i.v. injections of AAVrh10 *GALT*. Different dosages at (1 × 10^12^, 1 × 10^13^, and 1 × 10^14^ viral genomes/kg body weight) were used at designated experiments. All of the mice were randomized based on weight for each experiment. At 1, 8, and 16 weeks postinjection, the mice were evaluated for labeled galactose oxidation. At 24 weeks postinjection, mice were sacrificed, and the livers were harvested immediately to analyze the GALT protein expression, enzyme activity, and Gal-1P accumulation.

### Galactose oxidation measurement

To evaluate the whole-body galactose oxidation in WT, *GalT* KO, and *GalT* heterozygous mice, a 1-^13^C breath test was performed. We purchased 1-^13^C labeled galactose from Cambridge Isotope Laboratories (Andover, MA). Mice were injected with an i.p. dose of 40 μL of 5 mg/mL [1-^13^C]-galactose dissolved in 0.9% saline and quickly relocated to individual metabolic chambers through which room air was constitutively circulated (∼1 L/min) except during breath collection periods as shown in Figure 1. Before each breath collection period, the metabolic chambers were flushed with CO_2_-free air (∼5 L/min) for 10 s to ensure all of the sampled CO_2_ was generated by the mouse and not derived from ambient air.^44^ Every 15 min for the next 2 h, the metabolic chambers were periodically sealed for 3 min to allow the CO_2_ to increase to ∼1%. A 20-mL subsample of the gas inside the metabolic chamber was removed using a gas-tight syringe and transferred into a Exetainer 12-mL coated vial 439W/NP (Labco, Lampeter, UK) at room temperature until the analysis. Baseline breath samples were collected before galactose administration.

### ^13^CO_2_ measurement in breath samples

The δ^13^C value of the CO_2_ in the breath samples was measured using a nondispersive infrared ^13^C analyzer following published protocols.^45^^,^^46^^,^^47^ Internal calibrations were conducted before each round of measurements, and vials containing externally validated laboratory standard gases were run in triplicate in series for every n = 12 breath samples. δ^13^C values are reported in terms of ^13^C_VPDB_^48^^,^^49^ and all of the measurements were made within 4 weeks of breath collection.^50^

### Gal-1P assay

Intracellular gal-1P level was assayed by the alkaline phosphatase coupled assay method previously described.^51^ The liver tissues from both treated *GalT KO* and untreated WT control were homogenized using a microcentrifuge pestle in 300 μL of ice-cold hypotonic buffer containing 25 mM Tris-HCl (pH 7.4), 25 mM NaCl, 0.5 mM EDTA, and protease inhibitor cocktail (Roche, Indianapolis, IN) and centrifuged for 20 min at 16,000 × *g* and 4°C. To the clear supernatant 3% perchloric acid was added, and the precipitated protein was removed by centrifugation for 20 min at 16,000 × *g* and 4°C. The gal-1P concentration was normalized to protein concentration.

### GALT enzyme activity assay

GALT activity was determined as described previously.^23^ In brief, 30 μg liver homogenate was incubated with 40 mM Tris-HCl pH 8.0, 40 μM DTT, 6 mM gal-1P, 125 mM glycine, and 1.5 mM UDP at 37°C for 5 min before starting the enzyme reaction by adding gal-1P (60 mM) for 30 min at 37°C. GALT enzyme reactions were terminated by adding 150 μL of ice-cold 3.3% (w/v) tricarboxylic acid and vortexing. After centrifugation at 12,000 × *g* for 3 min at 4°C, the supernatant was transferred to a new set of tubes. The mixture was neutralized by adding 8 μL ice-cold 5 M potassium carbonate. The samples were kept on ice for 10 min and centrifuged at 12,000 × *g* for 3 min at 4°C. Chromatographic separation and quantification were accomplished with a high-performance liquid chromatography (HPLC) system equipped with a quaternary pump, an autosampler, a thermostated column compartment, a Hypercarb HPLC column (100 mm × 2.1 mm, particle size 3 μm), and a diode array detector using UV detection at 260 nm, all used according to the manufacturers’ recommendations. Mobile phase A was 20 mM ammonium acetate and 0.1% ammonia, and mobile phase B was 50/50 mobile phase A/acetonitrile. The chromatographic conditions were t = 0 min, 10%B; t = 10 min, 50%B; t = 10.1 min, 80%B; t = 14 min, 80%B; t = 14.1 min, 10%B. The flow rate was set to 0.4 mL/min and the column temperature was set to 60°C.

### Western blot analysis

The western blotting was conducted according to the method described previously.^23^ Protein lysates from the liver tissues were prepared by mechanical disruption in hypotonic buffer (25 mM NaCl, 0.5 mM EDTA, 25 mM Tris HCl pH 7.2) with complete protease and phosphatase inhibitors (Roche) at 4°C. The cell debris was removed by centrifugation at 13,000 rpm for 15 min at 4°C. Pierce BCA protein estimation kit (Thermo Fisher Scientific, Whaltham, MA) was used to determine the total protein content. We resolved 20 μg of the total protein by 12% SDS-PAGE before being transferred to a nitrocellulose membrane. Membranes were incubated for 2 h at room temperature with primary antibodies: polyclonal rabbit anti-*GALT* (Abcam, Cambridge, UK) or antiglyceraldehyde 3-phosphate dehydrogenase (GAPDH; Cell Signaling Technology, Danvers, MA), which served as loading control. Primary antibodies were detected with infrared dye-conjugated secondary antibodies and visualized by Odyssey Image Analyzer (Li-Cor Biotechnology, Lincoln, NE). Quantitative analysis of the fluorescence signals was performed by Empiria Studio Lite software (Li-Cor Biotechnology), and the results were normalized to the corresponding GAPDH abundance detected from the same blot. Student’s t test was used to determine statistical significance of the results.

### GALT protein quantification by liquid chromatography-tandem mass spectrometry (LC-MS/MS)

Liver tissue samples were homogenized in a buffer containing 8 M urea and 100 mM ammonium bicarbonate using a Precellys Evolution. The homogenate was centrifuged at 18,000 × *g* at 4 °C for 20 min. Supernatants were removed and assayed for protein concentration; 100 μg each were taken through a trypsin digestion protocol. Each sample was spiked with isotopically labeled signature peptide (ALPEVHYHLGQK and VMCGHPWSDVTLPLMSVPEIR, natural C and N atoms on arginine and lysine are fully replaced by ^13^C and ^15^N isotopes, respectively) as internal standard. Standard curves were generated using corresponding light signature peptides with flanking regions to account for digestion efficiency (ERLRALPEVHYHLGQKDRET and GVCKVMCFHPWSDVTLPLMSVPEIRAVVD for alkaline phosphatase and VMC [VMCGHPWSDVTLPLMSVPEIR] peptides, respectively). Denatured samples were reduced with 10 mM Tris(2-carboxyethyl)phosphine at 37°C for 1 h. Then, reduced samples were alkylated with 0.1 M iodoacetamide at room temperature for 1 h in the dark. Trypsin digestion was performed overnight at 37°C. Samples were cleaned up using a SOLA horseradish peroxidase solid-phase extraction. Eluted samples were dried and reconstituted in water and 0.1% formic acid.

LC-MS/MS analysis was performed on a Q Exactive Plus mass spectrometer (Thermo Fisher) coupled to an Easy-nLC 1200. Peptide digests were pressure loaded onto an Easy-Spray PepMap Neo 2 μm C18 75 μm × 150 mm nano flow column. The peptides were eluted using a gradient of 2%–45% buffer B in buffer A (buffer A: 98% water, 2% acetonitrile, 0.1% formic acid; buffer B: 10% water, 90% acetonitrile, 0.1% formic acid). The flow rate through the column was set to 0.3 μL/min, with a column temperature of 60°C. The parallel reaction monitoring (PRM)-MS data were collected for GALT-specific tryptic peptides. A static modification of +57.02146 on cysteine was specified to account for alkylation by iodoacetamide. PRM windows were scheduled for 5 min at a 17,500 resolution. As a minimum, the sampling rate resulted in six points across the curve. Absolute protein quantitation was calculated using the ratio of light-to-heavy peptide abundance values in Skyline.

### Statistical analysis

Microsoft Excel and GraphPad Prism 9 software were used to analyze data. The Student’s t test and one-way ANOVAs test were used for comparisons between the groups.

Whole-animal galactose oxidation was compared using ANOVAs and Pearson correlation tests followed by Tukey HSD post hoc analyses using Systat version 13.2.

## Data and code availability

Data supporting the studies presented in this paper can be made available by request to the corresponding authors.

## References

[1] Isselbacher K.J., Anderson E.P., Kurahashi K., Kalckar H.M. (1956). Congenital galactosemia, a single enzymatic block in galactose metabolism. Science.

[2] Fridovich-Keil J.L. (2006). Galactosemia: the good, the bad, and the unknown. J. Cell. Physiol..

[3] Tyfield L., Reichardt J., Fridovich-Keil J., Croke D.T., Elsas L.J., Strobl W., Kozak L., Coskun T., Novelli G., Okano Y. (1999). Classical galactosemia and mutations at the galactose-1-phosphate uridyl transferase (GALT) gene. Hum. Mutat..

[4] Berry G.T., Segal S., Gitzelmann R., Fernandes J., Saudubray M., van den Berghe G., Walter J.H. (2006). Inborn Metabolic Diseases - Diagnosis and Treatment.

[5] Segal S., Berry G.T., Scriver C.R., Beaudet A.L., Sly W.S., Valle D. (1995). The Metabolic Basis of Inherited Diseases.

[6] Leloir L.F. (1951). The enzymatic transformation of uridine diphosphate glucose into a galactose derivative. Arch. Biochem. Biophys..

[7] Gitzelmann R. (1995). Galactose-1-phosphate in the pathophysiology of galactosemia. Eur. J. Pediatr..

[8] Lai K., Langley S.D., Khwaja F.W., Schmitt E.W., Elsas L.J. (2003). GALT deficiency causes UDP-hexose deficit in human galactosemic cells. Glycobiology.

[9] Kaye C.I., Accurso F., La Franchi S., Lane P.A., Hope N., Sonya P., G Bradley S., Michele A L.P., Committee on Genetics (2006). Newborn screening fact sheets. Pediatrics.

[10] Berry G.T., Moate P.J., Reynolds R.A., Yager C.T., Ning C., Boston R.C., Segal S. (2004). The rate of de novo galactose synthesis in patients with galactose-1-phosphate uridyltransferase deficiency. Mol. Genet. Metabol..

[11] Berry G.T., Nissim I., Lin Z., Mazur A.T., Gibson J.B., Segal S. (1995). Endogenous synthesis of galactose in normal men and patients with hereditary galactosaemia. Lancet.

[12] Waggoner D., Buist N.R.M. (1993). Long-term complications in treated galactosemia - 175 U.S. cases. Int. Pediatr. J. Miami Child.

[13] Waggoner D.D., Buist N.R., Donnell G.N. (1990). Long-term prognosis in Galactosemia: results of a survey of 350 cases. J. Inherit. Metab. Dis..

[14] Batey L.A., Welt C.K., Rohr F., Wessel A., Anastasoaie V., Feldman H.A., Guo C.Y., Rubio-Gozalbo E., Berry G., Gordon C.M. (2012). Skeletal health in adult patients with classic galactosemia. Osteoporosis Int...

[15] Panis B., Forget P.P., van Kroonenburgh M.J.P.G., Vermeer C., Menheere P.P., Nieman F.H., Rubio-Gozalbo M.E. (2004). Bone metabolism in galactosemia. Bone.

[16] Rubio-Gozalbo M.E., Hamming S., van Kroonenburgh M.J.P.G., Bakker J.A., Vermeer C., Forget P.P. (2002). Bone mineral density in patients with classic galactosaemia. Arch. Dis. Child..

[17] Berry G.T. (2011). Is prenatal myo-inositol deficiency a mechanism of CNS injury in galactosemia?. J. Inherit. Metab. Dis..

[18] Bhat P.J. (2003). Galactose-1-phosphate is a regulator of inositol monophosphatase: a fact or a fiction?. Med. Hypotheses.

[19] Charlwood J., Clayton P., Keir G., Mian N., Winchester B. (1998). Defective galactosylation of serum transferrin in galactosemia. Glycobiology.

[20] Coss K.P., Hawkes C.P., Adamczyk B., Stöckmann H., Crushell E., Saldova R., Knerr I., Rubio-Gozalbo M.E., Monavari A.A., Rudd P.M., Treacy E.P. (2014). N-glycan abnormalities in children with galactosemia. J. Proteome Res..

[21] Ornstein K.S., McGuire E.J., Berry G.T., Roth S., Segal S. (1992). Abnormal galactosylation of complex carbohydrates in cultured fibroblasts from patients with galactose-1-phosphate uridyltransferase deficiency. Pediatr. Res..

[22] Prestoz L.L., Couto A.S., Shin Y.S., Petry K.G. (1997). Altered follicle stimulating hormone isoforms in female galactosaemia patients. Eur. J. Pediatr..

[23] Balakrishnan B., An D., Nguyen V., DeAntonis C., Martini P.G.V., Lai K. (2020). Novel mRNA-Based Therapy Reduces Toxic Galactose Metabolites and Overcomes Galactose Sensitivity in a Mouse Model of Classic Galactosemia. Mol. Ther..

[24] Brophy M.L., Stansfield J.C., Ahn Y., Cheng S.H., Murphy J.E., Bell R.D. (2022). AAV-mediated expression of galactose-1-phosphate uridyltransferase corrects defects of galactose metabolism in classic galactosemia patient fibroblasts. J. Inherit. Metab. Dis..

[25] Berry G.T., Nissim I., Mazur A.T., Elsas L.J., Singh R.H., Klein P.D., Gibson J.B., Lin Z., Segal S. (1995). In vivo oxidation of [13C]galactose in patients with galactose-1-phosphate uridyltransferase deficiency. Biochem. Mol. Med..

[26] Berry G.T., Reynolds R.A., Yager C.T., Segal S. (2004). Extended [13C]galactose oxidation studies in patients with galactosemia. Mol. Genet. Metabol..

[27] Berry G.T., Singh R.H., Mazur A.T., Guerrero N., Kennedy M.J., Chen J., Reynolds R., Palmieri M.J., Klein P.D., Segal S., Elsas L.J. (2000). Galactose breath testing distinguishes variant and severe galactose-1-phosphate uridyltransferase genotypes. Pediatr. Res..

[28] Guerrero N.V., Singh R.H., Manatunga A., Berry G.T., Steiner R.D., Elsas L.J. (2000). Risk factors for premature ovarian failure in females with galactosemia. J. Pediatr..

[29] Lai K., Langley S.D., Singh R.H., Dembure P.P., Hjelm L.N., Elsas L.J. (1996). A prevalent mutation for galactosemia among black Americans. J. Pediatr..

[30] Webb A.L., Singh R.H., Kennedy M.J., Elsas L.J. (2003). Verbal dyspraxia and galactosemia. Pediatr. Res..

[31] Sabnis S., Kumarasinghe E.S., Salerno T., Mihai C., Ketova T., Senn J.J., Lynn A., Bulychev A., McFadyen I., Chan J. (2018). A Novel Amino Lipid Series for mRNA Delivery: Improved Endosomal Escape and Sustained Pharmacology and Safety in Non-human Primates. Mol. Ther..

[32] Rubio-Gozalbo M.E., Haskovic M., Bosch A.M., Burnyte B., Coelho A.I., Cassiman D., Couce M.L., Dawson C., Demirbas D., Derks T. (2019). The natural history of classic galactosemia: lessons from the GalNet registry. Orphanet J. Rare Dis..

[33] Waisbren S.E., Potter N.L., Gordon C.M., Green R.C., Greenstein P., Gubbels C.S., Rubio-Gozalbo E., Schomer D., Welt C., Anastasoaie V. (2012). The adult galactosemic phenotype. J. Inherit. Metab. Dis..

[34] Cuthbert C., Klapper H., Elsas L. (2008). Diagnosis of inherited disorders of galactose metabolism. Curr. Protoc. Hum. Genet..

[35] Copenhaver J.H., Bausch L.C., Fitzgibbons J.F. (1969). A fluorometric procedure for estimation of galactose-1-phosphate uridylyltransferase activity in red blood cells. Anal. Biochem..

[36] Inouye T., Nadler H.L., Hsia Y.Y. (1968). Galactose-I-phosphate uridyltransferase in red and white blood cells. Clin. Chim. Acta.

[37] Airey C.M., Price D.E., Kemp J.V., Perkins C.M., Wales J.K. (1989). The effect of aldose reductase inhibition on erythrocyte polyols and galactitol accumulation in diabetic patients. Diabet. Med..

[38] Murata M., Ohta N., Fujisawa S., Tsai J.Y., Sato S., Akagi Y., Takahashi Y., Neuenschwander H., Kador P.F. (2002). Selective pericyte degeneration in the retinal capillaries of galactose-fed dogs results from apoptosis linked to aldose reductase-catalyzed galactitol accumulation. J. Diabet. Complicat..

[39] Robertson A., Singh R.H., Guerrero N.V., Hundley M., Elsas L.J. (2000). Outcomes analysis of verbal dyspraxia in classic galactosemia. Genet. Med..

[40] Manoli I., Pass A.R., Harrington E.A., Sloan J.L., Gagné J., McCoy S., Bell S.L., Hattenbach J.D., Leitner B.P., Duckworth C.J. (2021). Correction to: 1-(13)C-propionate breath testing as a surrogate endpoint to assess efficacy of liver-directed therapies in methylmalonic acidemia (MMA). Genet. Med..

[41] Manoli I., Pass A.R., Harrington E.A., Sloan J.L., Gagné J., McCoy S., Bell S.L., Hattenbach J.D., Leitner B.P., Duckworth C.J. (2021). 1-(13)C-propionate breath testing as a surrogate endpoint to assess efficacy of liver-directed therapies in methylmalonic acidemia (MMA). Genet. Med..

[42] Rasmussen S.A., Daenzer J.M.I., Fridovich-Keil J.L. (2021). A pilot study of neonatal GALT gene replacement using AAV9 dramatically lowers galactose metabolites in blood, liver, and brain and minimizes cataracts in GALT-null rat pups. J. Inherit. Metab. Dis..

[43] Tang M., Siddiqi A., Witt B., Yuzyuk T., Johnson B., Fraser N., Chen W., Rascon R., Yin X., Goli H. (2014). Subfertility and growth restriction in a new galactose-1phosphate uridylyltransferase (GALT) – deficient mouse model. Eur. J. Hum. Genet..

[44] McCue M.D. (2023). CO(2) scrubbing, zero gases, Keeling plots, and a mathematical approach to ameliorate the deleterious effects of ambient CO(2) during (13) C breath testing in humans and animals. Rapid Commun. Mass Spectrom..

[45] Hatle J.D., Awan A., Nicholas J., Koch R., Vokrri J.R., McCue M.D., Williams C.M., Davidowitz G., Hahn D.A. (2017). Life-extending dietary restriction and ovariectomy each increase leucine oxidation and alter leucine allocation in grasshoppers. Exp. Gerontol..

[46] McCue M.D., Passement C.A., Rodriguez M. (2015). The magnitude of the naturally occurring isotopic enrichment of 13C in exhaled CO2 is directly proportional to exercise intensity in humans. Comp. Biochem. Physiol. Mol. Integr. Physiol..

[47] McCue M.D., Welch K.C. (2016). (13)C-Breath testing in animals: theory, applications, and future directions. J. Comp. Physiol. B.

[48] Slater C., Preston T., Weaver L.T. (2001). Stable isotopes and the international system of units. Rapid Commun. Mass Spectrom..

[49] Welch K.C., Péronnet F., Hatch K.A., Voigt C.C., McCue M.D. (2016). Carbon stable-isotope tracking in breath for comparative studies of fuel use. Ann. N. Y. Acad. Sci..

[50] McCue M.D., Sivan O., McWilliams S.R., Pinshow B. (2010). Tracking the oxidative kinetics of carbohydrates, amino acids and fatty acids in the house sparrow using exhaled 13CO2. J. Exp. Biol..

[51] Balakrishnan B., Chen W., Tang M., Huang X., Cakici D.D., Siddiqi A., Berry G., Lai K. (2016). Galactose-1 phosphateuridylyltransferase (GalT) gene: a novel positive regulator of the PI3K/Akt signaling pathway in mouse fibroblasts. Biochem. Biophys. Res. Commun..

